# Complete Genome Sequence of MIDG2331, a Genetically Tractable Serovar 8 Clinical Isolate of *Actinobacillus pleuropneumoniae*

**DOI:** 10.1128/genomeA.01667-15

**Published:** 2016-01-28

**Authors:** Janine T. Bossé, Roy R. Chaudhuri, Yanwen Li, Leon G. Leanse, Roberto Fernandez Crespo, Paul Coupland, Matthew T. G. Holden, Denise M. Bazzolli, Duncan J. Maskell, Alexander W. Tucker, Brendan W. Wren, Andrew N. Rycroft, Paul R. Langford

**Affiliations:** a Department of Medicine, Section of Paediatrics, Imperial College London, London, United Kingdom; b Department of Veterinary Medicine, University of Cambridge, Cambridge, United Kingdom; c The Wellcome Trust Sanger Institute, Cambridge, United Kingdom; d Departamento de Microbiologia, Laboratório de Genética Molecular de Micro-organismos, Universidade Federal de Viçosa, Viçosa, Brazil; e Faculty of Infectious & Tropical Diseases, London School of Hygiene and Tropical Medicine, London, United Kingdom; f Department of Pathology and Pathogen Biology, The Royal Veterinary College, Hatfield, United Kingdom

## Abstract

We report here the complete annotated genome sequence of a clinical serovar 8 isolate *Actinobacillus pleuropneumoniae* MIDG2331. Unlike the serovar 8 reference strain 405, MIDG2331 is amenable to genetic manipulation via natural transformation as well as conjugation, making it ideal for studies of gene function.

## GENOME ANNOUNCEMENT

*Actinobacillus pleuropneumoniae* is a respiratory tract pathogen of swine that causes significant economic losses worldwide. Fifteen serovars of *A. pleuropneumoniae* vary in geographic distribution (1). Serovar 8, found in many countries around the world (1), is the predominant serovar in the United Kingdom (2) and southeastern Brazil (3). A United Kingdom clinical serovar 8 isolate, MIDG2331, was shown to be highly competent for natural transformation (4) and amenable to conjugal transfer of plasmids (5). This isolate is ideally suited for facile construction of gene deletions as well as complementation using shuttle vectors such as pMC-Express and pMK-Express (6).

The complete genome of sequence of MIDG2331 was determined using a combination of Illumina GAII and PacBio RS II platforms. Illumina sequencing yielded a total of 1,121,724 read pairs of 2 × 76 bp, of which 1,118,490 were retained after adapter trimming using Cutadapt version 1.8.1 (7). PacBio sequencing yielded 13,273 circular consensus sequence (ccs) reads, with a mean length of 2,861.4 bp (longest 13,273 bp), and 15,276 corrected long reads, with a mean length of 5,997.7 bp (longest 17,412 bp).

Individual assembly of the Illumina and PacBio datasets using SPAdes (8) and HGAP (9), respectively, resulted in multiple-contig assemblies (65 Illumina contigs; 2 PacBio contigs). A hybrid SPAdes assembly using both Illumina and PacBio datasets resolved into a single contig that was circularized using Circlator (10) and corrected based on the PacBio ccs reads using Quiver (9), giving a final assembly of 2,337,633 bp. Automated sequence annotation was performed using Prokka version 1.11 (11), which predicted 2,174 putative open reading frames (ORFs), 63 tRNAs, and 6 rRNA operons. The average GC content of the genome is 41.1%, similar to other members of the *Pasteurellaceae*.

No plasmid DNA was found in MIDG2331, although plasmids have been described in other strains of this species. However, we have identified a 56-kb sequence in the genome showing a high degree of similarity to previously reported Integrative Conjugative Elements of the ICE*Hin1056* family (12, 13).

We previously characterized the reference strains of *A. pleuropneumoniae* with regard to competence for natural transformation. Serovars 1, 3, 4, 5, and 8 all showed low frequencies of transformation (10^−8^ to 10^−9^), whereas the serovar 15 reference strain, HS143, had a transformation frequency of 10^−4^ (14). Subsequently, we identified MIDG2331 as highly competent, with a transformation frequency of 10^−5^ (4). The genome of MIDG2331 contains a full set of known competence genes (14) as well as 770 copies of the 9-bp sequence ACAAGCGGT, a DNA uptake signal sequence (USS) which is highly overrepresented in the genomes of the *Apl* subclade of the *Pasteurellaceae* (15). Although the serovar 5 L20 genome contains the same complete set of competence genes and 742 copies of the *A. pleuropneumoniae* USS (16), natural transformation is not efficient in this strain. More detailed analysis of the MIDG2331 genome, the first from a highly competent *A. pleuropneumoniae* isolate, may help identify factor(s) which contribute to transformation efficiency in this bacterium.

### Nucleotide sequence accession number.

The complete genome sequence of MIDG2331 was deposited in GenBank under the accession number LN908249.
